# The lung microbiota in early rheumatoid arthritis and autoimmunity

**DOI:** 10.1186/s40168-016-0206-x

**Published:** 2016-11-17

**Authors:** Jose U. Scher, Vijay Joshua, Alejandro Artacho, Shahla Abdollahi-Roodsaz, Johan Öckinger, Susanna Kullberg, Magnus Sköld, Anders Eklund, Johan Grunewald, Jose C. Clemente, Carles Ubeda, Leopoldo N. Segal, Anca I. Catrina

**Affiliations:** 1 Division of Rheumatology, NYU School of Medicine, New York, NY USA; 2 Rheumatology Unit, Department of Medicine, Karolinska Institutet, Karolinska University Hospital, Stockholm, Sweden; 3 Institute for Research in Public Health, Valencia, Spain; 4 Respiratory Medicine Unit, Department of Medicine Solna, Center for Molecular Medicine, Karolinska Institutet, Stockholm, Sweden; 5 Department of Genetics and Genomic Sciences, Icahn Institute for Genomics and Multiscale Biology, Icahn School of Medicine at Mount Sinai, New York, NY USA; 6 Division of Pulmonary and Critical Care Medicine, NYU School of Medicine, New York, NY USA

## Abstract

**Background:**

Airway abnormalities and lung tissue citrullination are found in both rheumatoid arthritis (RA) patients and individuals at-risk for disease development. This suggests the possibility that the lung could be a site of autoimmunity generation in RA, perhaps in response to microbiota changes. We therefore sought to test whether the RA lung microbiome contains distinct taxonomic features associated with local and/or systemic autoimmunity.

**Methods:**

16S rRNA gene high-throughput sequencing was utilized to compare the bacterial community composition of bronchoalveolar lavage fluid (BAL) in patients with early, disease-modifying anti-rheumatic drugs (DMARD)-naïve RA, patients with lung sarcoidosis, and healthy control subjects. Samples were further assessed for the presence and levels of anti-citrullinated peptide antibodies (including fine specificities) in both BAL and serum.

**Results:**

The BAL microbiota of RA patients was significantly less diverse and abundant when compared to healthy controls, but similar to sarcoidosis patients. This distal airway dysbiosis was attributed to the reduced presence of several genus (i.e., *Actynomyces* and *Burkhordelia*) as well as reported periodontopathic taxa, including *Treponema*, *Prevotella*, and *Porphyromonas*. While multiple clades correlated with local and systemic levels of autoantibodies, the genus *Pseudonocardia* and various related OTUs were the only taxa overrepresented in RA BAL and correlated with higher disease activity and erosions.

**Conclusions:**

Distal airway dysbiosis is present in untreated early RA and similar to that detected in sarcoidosis lung inflammation. This community perturbation, which correlates with local and systemic autoimmune/inflammatory changes, may potentially drive initiation of RA in a proportion of cases.

**Electronic supplementary material:**

The online version of this article (doi:10.1186/s40168-016-0206-x) contains supplementary material, which is available to authorized users.

## Background

Rheumatoid arthritis (RA) is currently considered a complex, polygenic, and multifactorial disease. Novel concepts in its etiopathogenesis posit that, in the right genetic background (i.e., “shared epitope” alleles), a proportion of these individuals lose tolerance against self-peptides and enter a prolonged autoimmune phase, characterized by the production of circulating autoantibodies [(i.e., rheumatoid factor (RF) and anti-citrullinated peptide antibodies (ACPAs)] [1]. These antibodies, however, arise in the circulation in the absence of major synovial pathology, which has led to a renovated search for extra-articular epigenetic and environmental triggers of disease [2]. Among the latter, interest in the role of microorganisms residing in mucosal sites (i.e., microbiome) and the associated host immune response has reemerged [3]. Multiple lines of investigation utilizing models of inflammatory arthritis have demonstrated that, in the absence of bacteria, animals are typically spared from disease and that perturbation in the bacterial community composition (dysbiosis), rather than the presence of pathogens, is sufficient for the development of joint disease [3–5]. Others and we have reported on an analogous dysbiotic process in the intestinal and oral microbiome of RA and psoriatic arthritis (PsA) patients [6–8].

The lung has also been implicated as a potential site of extra-articular autoimmune generation [9]. This is based on the epidemiological association between tobacco smoking and RA, the finding of distal airway lesions in early disease and at-risk individuals, and the presence of ACPAs in induced sputum of RA patients (even in the absence of circulating autoantibodies) and lung tissue [10–13]. Enrichment of the lower airway microbiome with taxa commonly found in the upper airways is associated with local markers of ongoing inflammation [14, 15]. However, the potential contribution of the lung microbiome in early disease has never been investigated. Here, we performed research bronchoscopies to characterize the microbiota composition in the bronchoalvolar lavage (BAL) of early-untreated RA to compare it with that of a well-defined inflammatory lung disease (sarcoidosis) and to relate it to local and systemic immune response.

## Methods

### Patients

Patients diagnosed with RA according to 1987 ACR criteria [16] and included in the LUng investigation in early RA (LURA) study at Karolinska University Hospital in Stockholm [13] underwent research bronchoscopy, with retrieval of BAL as described in previous studies [11]. Newly diagnosed pulmonary sarcoidosis patients and healthy volunteers were enrolled as controls. In parallel, newly diagnosed sarcoidosis patients and healthy volunteers were enrolled as controls by the same investigators as those performing bronchoscopy on RA patients (SK, MS, and AE). All subjects were enrolled at the Karolinska University Hospital in Stockholm.

Detailed demographic characteristics of the patients are given in Table 1. None of the participants reported recent antibiotic usage (<3 months).

**Table 1:** Clinical and Demographic characteristics of participants

	Rheumatoid arthritis	Sarcoidosis	Healthy controls	*P* value
Number of subjects, *n*	20	10	28	
Female, *n* (%)	8 (40 %)	3 (30 %)	14 (50 %)	0.52
Age (years), median (range)	59 (28–76)	40 (30–63)	28 (19–50)	<0.001
ACPA positive, *n* (%)	16 (80 %)	0 (0%)	1^b^ (4 %)	<0.001
Smoking				0.005
Non-smokers, *n* (%)	4 (20 %)	4 (40 %)	14 (50 %)	
Ex-smokers, *n* (%)	7 (35 %)	4 (40 %)	0 (0 %)	
Current-smokers, *n* (%)	9 (45 %)	2 (20 %)	14 (50 %)	
Disease activity				
DAS28, median (range)	4.42 (2.97–6.69)	n.a.	n.a.	n.a.
CRP, median (range)	10.5 (1–54)	n.a.	n.a.	n.a.
ESR, median (range)	20 (4–77)	n.a.	n.a.	n.a.
Bone erosion, *n* (%)	7 (35 %)	n.a.	n.a.	n.a.
Löfgren syndrome, *n* (%)	n.a.	5 (50 %)	n.a.	n.a.
Pulmonary function test				
VC (%), median (range)	104 (74–136)	85.5 (64–93)^c^	n.a.	0.002
FVC (%), median (range)	106 (77–138)	86 (66–90)^d^	107 (84–135)	0.004
FEV_1_ (%), median (range)	96 (49–126)	76.5 (64–95)^c^	103 (82–122)	0.006
*D*_LCO_ (%), median (range)	80 (40–135)	75 (66–84)^d^	n.a.	0.43
Bronchoscopy				
BAL recovery (%), median (range)	58 (30–85)	68 (45–76)	69 (25–80)	0.07
BAL cell concentration (10^6^ cells l ^−1^), median (range)	160.5 (78.7–710)	172.75 (80–388)	153.25 (14.3–987.2)	0.90
Macrophages (%), median (range)	91.4 (56.4–97.4)	69.9 (46.6–94.8)	93.8 (71.8–99)^a^	<0.001
Lymphocytes (%), median (range)	7.2 (2–34)	27.6 (4.2–49.3)	4.2 (0.4–14.8)^a^	<0.001
Neutrophils (%), median (range)	1.5 (0.2–7)	1.6 (0.5–4)	0.6 (0.2–18.8)^a^	0.05
Eosinophils (%), median (range)	0 (0–5)	0.1 (0–3.7)	0.2 (0–0.8)^a^	0.21

Between groups comparisons were made with one-way ANOVA (normally distributed continuous variables), the Kruskal-Wallis test (non-normally distributed continuous variables), and the *χ* ^2^ test (categorical variables)

Pulmonary function test (values show % of predicted): *VC* vital capacity, *FVC* forced vital capacity, *FEV1* forced expiratory volume in 1 s, *DLCO* diffusing capacity of the lung for CO

*BAL* bronchoalveolar lavage

^a^1 missing data

^b^3 missing data

^c^4 missing data

^d^5 missing data

RA patients had symptom duration of less than a year (median 6 months, range 3–12 months) and a median age of 59 years (range 28–76 years). None of them have ever received any oral glucocorticoids, disease-modifying anti-rheumatic drugs (DMARDs), or biologic drugs. All included RA patients had imaging of the thorax. Parenchymal changes were defined as the presence of nodules larger than 3 mm, ground-glass opacities, opacities, fibrosis, and emphysema. Twelve out of 20 RA patients had at least one of these abnormalities on CT double blind evaluation.

All sarcoidosis patients had typical clinical signs and symptoms of sarcoidosis (including cough, fever, chest pain, and fatigue) and chest X-ray findings compatible with sarcoidosis. Diagnosis was set according to criteria established by WASOG guidelines, with biopsies showing non-caseating granuloma formation and/or through differential BALF cell counts showing an elevated BALF CD4/CD8 ratio (median 5.7; min-max 1.3–24.9), and by ruling out other causes of these observations. In six of the patients, CT scans were also performed in addition to chest radiography.

Patients with Löfgren’s syndrome were identified by acute onset of the disease with fever, erythema nodosum, and/or ankle arthritis, and bilateral hilar adenopathy with or without concomitant parenchymal infiltrates. Chest radiographic classification of patients with sarcoidosis showed that four had stage I (hilar lymphadenopathy), four were in stage II (pulmonary infiltrates with hilar lymphadenopathy), and two patients were in stage III (pulmonary infiltrates without hilar lymphadenopathy). All patients with Löfgren’s syndrome had chest radiographic stage I or II. None of the sarcoidosis patients had received any immunosuppressant therapy at the time of bronchoscopy.

Healthy controls, with a normal chest X-ray and a median age of 28 years (range 19–50), were concomitantly recruited through advertisement at the Lung Allergy Clinic, Karolinska University Hospital, Solna, Sweden. Bronchoscopies and lavage were done in a similar fashion as in RA and sarcoidosis patients. None of the healthy controls had clinically relevant airway infections or allergy symptoms at the time of bronchoscopy, and subjects diagnosed with asthma, COPD, other lung diseases, or other inflammatory conditions were excluded from the study. Written informed consent was obtained from all subjects, and the Regional Ethical Review Board in Stockholm approved the studies.

### Procedures

Bronchoscopy was performed as previously described [17, 18]. Whole unfractionated BAL samples were utilized for 16S sequencing analysis (see Additional file 1 for more details).

### Antibody assays

Anti-CCP2 antibodies in the serum and BAL were detected using the enzyme-linked immunosorbent assay (ELISA) (Euro-Diagnostica AB, Sweden) according to manufacturer’s instructions. Serum samples were analyzed for specific ACPA IgGs using a custom-made peptide microarray based on the ImmunoCAP ISAC system (PhaDia) described in detail earlier [19].

### Statistical analysis

In order to identify differentially abundant bacterial taxa among the three groups, we applied the Benjamini and Hochberg false discovery rate (FDR) test and/or the LefSe analytic method. For cross-sectional analyses of baseline characteristics and comparison of diversity indexes between groups, differences were evaluated using the two-tailed Student’s *t* test. The ANOSIM test was applied to the unweighted UniFrac distance matrix containing all analyzed samples in order to define if the overall structure of the microbiota was significantly different between the different groups. *P* values less than 0.05 were considered significant. Spearman’s correlation analyses were used to assess potentially clinically relevant associations on all taxa, irrespective of statistical threshold. The optimal Bayesian network structure was inferred through the “high climbing” algorithm implemented in the bnlearn R package (see Additional file 1 for more details and references).

## Results

### Patients

Detailed demographic characteristics of the patients and controls used in the study are given in Table 1. Compared to healthy and sarcoidosis patients, RA subjects were significantly older. Eighty percent of the RA patients were ACPA positive. In the spirometry test, sarcoid patients had significantly lower volume capacity (VC), forced vital capacity (FVC), and forced expiratory volume in 1 s (FEV_1_) compared to RA and healthy individuals. When comparing the BAL fluid content, the pulmonary sarcoidosis patients had significantly higher proportion of lymphocytes and hence lower proportion of macrophages, compared to RA and healthy.

### Distinct features of the lower airway microbiota

**Fig. 1:**
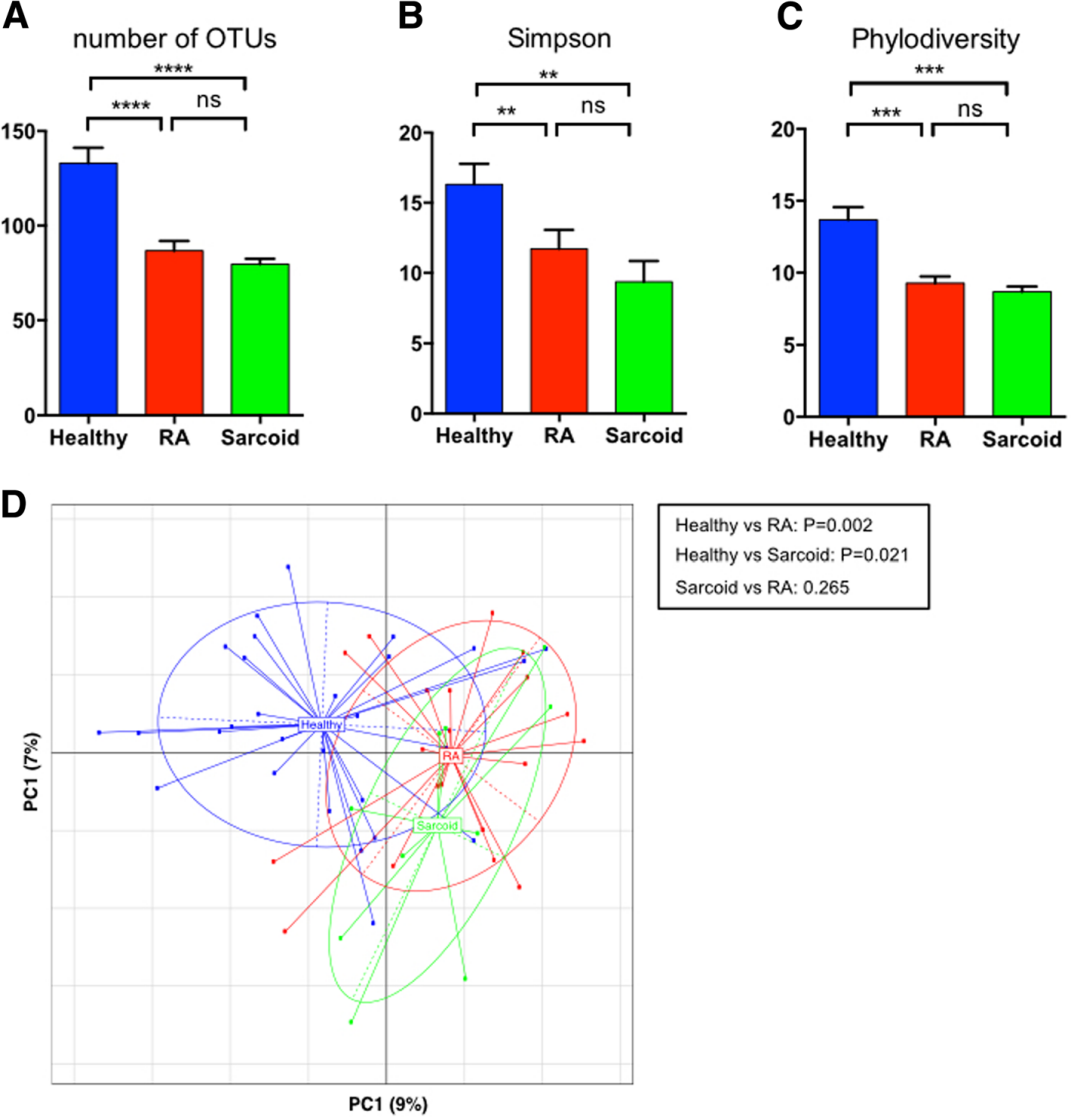
BAL microbiota richness and diversity. Alpha diversity was calculated using number of OTUs (**a**), Simpson diversity index (**b**), and the Faith’s phylodiversity index (**c**), all revealing significant differences between healthy subjects and sarcoid or RA. No significant differences were found between both patient groups. Beta diversity (**d**) demonstrated that RA and sarcoid samples clustered together and away from healthy controls. **P < 0.01; ***P < 0.001; ****P < 0.0001; *ns* non-significant

All BAL samples yielded 16S rRNA V4 gene sequences with a median depth of sequencing of 6365 reads per sample (IQR = 5,397–8061). When compared to healthy subjects, BAL microbial alpha-diversity was significantly reduced in both RA and sarcoidosis samples, as calculated by the total number of operational taxonomic units (OTUs) present, the Simpson diversity index, and the Faith’s phylodiversity index (Fig. 1a–c). Subsequently, we analyzed whether the overall structure of the microbiota of healthy samples differed from that of RA and sarcoidosis based on unweighted UniFrac distance. We further applied PCoA to cluster samples along orthogonal axes of maximal variance. As shown in Fig. 1d, β-diversity plots differentiated the lung microbiota of healthy individuals, as compared to RA or sarcoidosis patients (ANOSIM test; *P* = 0.002 and 0.021 for healthy vs RA and healthy vs sarcoidosis, respectively). However, no significant differences were found in β-diversity between RA and sarcoidosis patients. Further, β-diversity analysis did not differentiate smokers from non-smokers (*P* = n.s.; data not shown). Comparison of unweighted UniFrac distances between pairs of samples between different groups showed that the microbial community structure in RA and sarcoidosis patients was more closely related to each other than to healthy subjects (Additional file 2: Figure S1).

### RA and sarcoidosis BAL lack several taxa found in healthy individuals

**Fig. 2:**
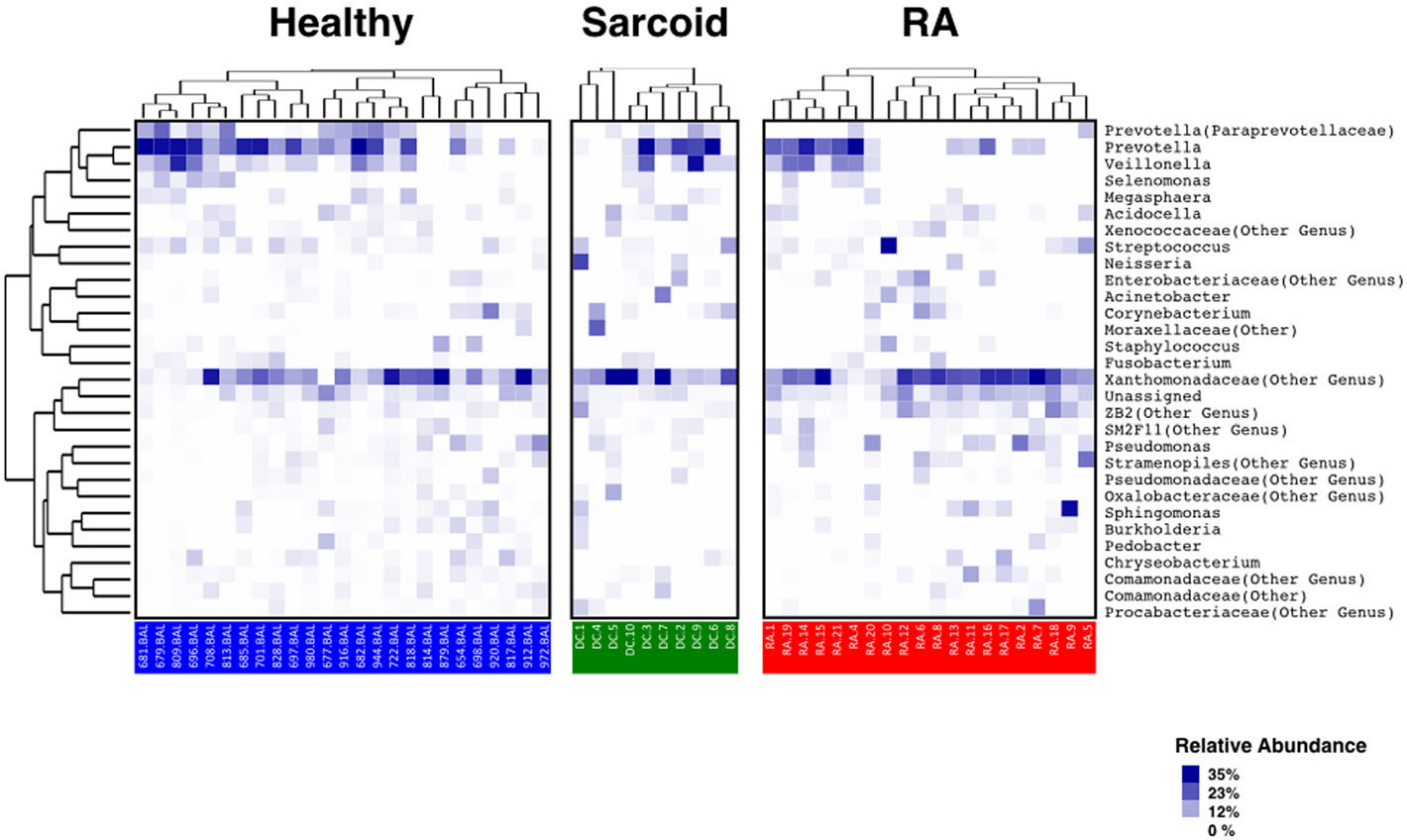
Taxonomic heatmap for each group. Characteristic relative abundance of various genus in healthy subjects (in *blue*), sarcoid (in *green*), and RA patients (in *red*). Each *column* represents a unique subject. On the *far right* are the most abundant genus found (mean > 0.5%) for all groups

**Fig. 3:**
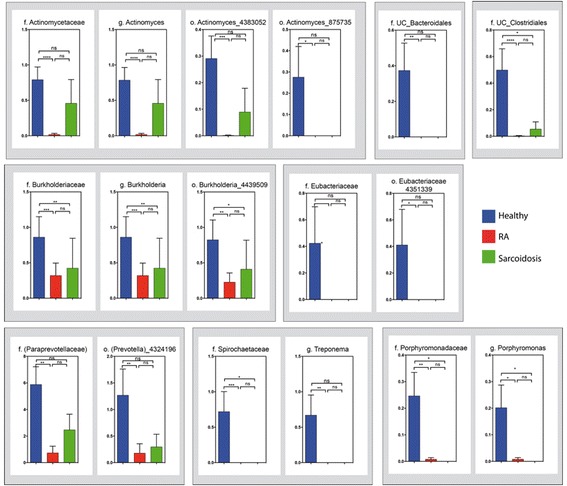
Relative abundance at the family, genus, and OTU levels. RA BAL samples showed a significant decrease in *Actynomyces* (and related OTUs), *Burkholderia* (and related OTUs), and *Treponema* (and *Prevotella*-related OTU), compared to healthy controls. A similar trend for *Burkholderia* and the family *Spirochaetaceae* was observed for sarcoid BAL. The relative abundance (and presence) of the genus *Porphyromonas* in healthy BAL was also higher than in RA and sarcoid samples. *P < 0.05; **P < 0.01; ***P < 0.001; ****P < 0.0001; *ns* non-significant

To further investigate which bacterial taxa were distinct among groups, we first analyzed the relative abundance of the most abundant taxa. As shown in Fig. 2, a heatmap revealed that most samples contained taxa belonging to the genera *Prevotella* and *Streptococcus*, and a genus annotated within the Xanthomonadaceae family. Other taxa (such as *Paraprevotellaceae*, *Chryseobacterium*, and *Burkhordelia*), while commonly found in healthy BAL, were less frequently present in samples from RA and sarcoid patients. We then applied Kruskall-Wallis followed by FDR correction and LefSe analysis (see Additional file 1) to better characterize these findings. Noticeably, the relative abundance of several microbial clades was decreased in RA and sarcoidosis samples compared to healthy controls (Fig. 3). Within these identified taxa, RA BAL samples had a decrease in the families *Burkholderiaceae*, *Actinomycetaceae*, *and Spirochaetaceae*. In fact, 68 % of the healthy control BAL contained *Actinomycetaceae* and 36 % had *Spirochaetaceae*, compared to 5 and 0 % in RA BAL, respectively (Fig. 3; *P* < 0.0001 for *Actinomycetaceae* and *P* = 0.0009 for *Spirochaetaceae*). At the genus level of taxonomic classification, *Burkholderia* was significantly decreased in both RA and sarcoidosis compared to controls, although there was no difference between disease groups (Fig. 3b). Contrary to our initial hypothesis that we would find a proportion of RA patients’ BAL containing *Porphyromonas* (potentially as a consequence of microaspiration of this periodontopathic genus), only 10 % of these samples contained sequences belonging to the genus. Curiously, however, 40% of the healthy controls BAL showed the presence of *Porphyromonas* (LDA score >3 vs RA and sarcoidosis; not achieving FDR; Additional file 3: Figure S2A, B). Similarly, the genus *Treponema* (also highly associated with periodontitis) was exclusively found in healthy subjects’ BAL (*P* < 0.01 vs RA; Fig. 3c and Additional file 3: Figure S2A). Comparable taxonomic differences were found between sarcoid and healthy BAL, with only a few genera being characteristic of sarcoid samples (Additional file 3: Figure S2B, C).

The use of state-of-the-art high-throughput sequencing platform allowed us for an in-depth analysis of the microbiota, including the characterization of the BAL OTUs that differentiate between groups. Several OTUs had a decreased relative abundance in RA BAL compared to healthy controls, including two belonging to the genus *Actinomyces*, one to *Burkhodelia* and another one to *Prevotella* (Fig. 3). Intriguingly, several of these OTUs were also decreased in sarcoidosis BAL, suggesting a potential common “inflammatory” lung microbiota signature for both conditions (Fig. 3; *P* = ns sarcoidosis vs RA). Although not achieving FDR, a few genus such as *Methylobacterium*, *Micrococcus*, and *Pseudonocardia* were more abundant in RA BAL compared to healthy controls (LDA > 3 vs healthy, Additional file 3: Figure S2). Moreover, an OTU aligned to *Pseudonocardia* (6305560) was the only BAL OTU overrepresented in the RA group when compared to both healthy controls and sarcoidosis patients (*P* < 0.01 and *P* < 0.05, respectively; although not achieving FDR; Additional file 4: Figure S3A).

### Local lung and systemic autoimmune generation in untreated, early RA is associated with characteristic BAL taxa

**Fig. 4:**
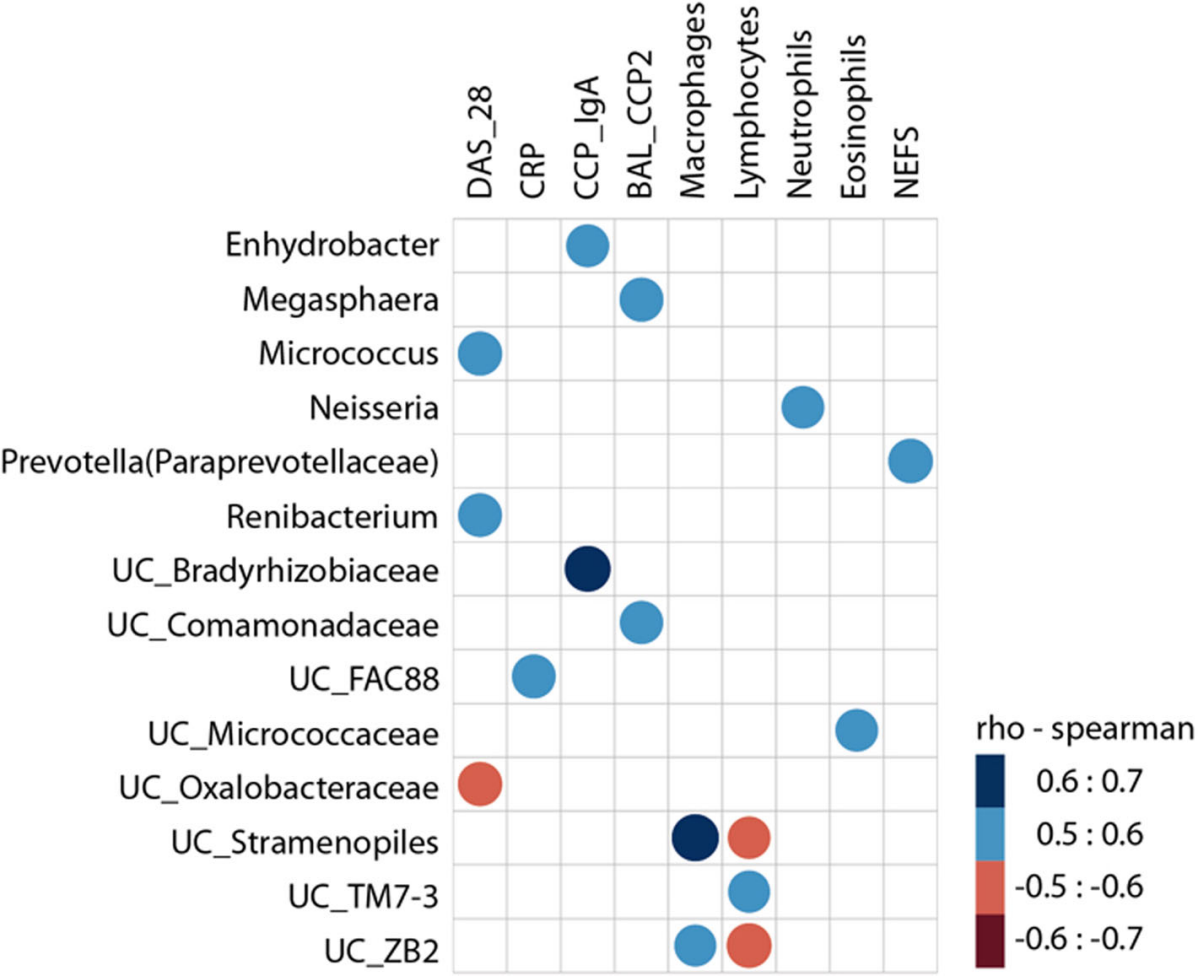
RA BAL microbiota correlations with local/systemic autoimmunity, inflammatory markers, and clinical disease activity. The relative abundance of BAL taxa (genus level) was assessed for correlations with disease activity score (DAS28), the levels of serum acute phase reactants and autoantibodies (including number of fine specificities), and BAL levels of anti-CCP2 and immune cells (%). The heat map shows the correlations between patient metadata and BAL microbiota at the genus level. *Circle sizes* and *color intensity* represent the magnitude of correlation. *Blue circles* = positive correlations; *red circles* = negative correlations. *CRP* C-reactive protein, *NEFS* number of ELISA ACPA fine specificities

To further investigate whether the observed alterations in BAL bacterial community composition in early RA patients was associated with phenotypic characteristics or metadata, we set out to describe the correlations between the relative abundance of taxa and (1) clinical disease activity (DAS28-ESR and CRP), (2) the local autoimmune response (BAL concentrations of ACPA and percentage of immune cells), and (3) the systemic immune response (serum concentrations of ACPA and RF) (Fig. 4 for genus; and Additional file 5: Figure S4 for OTUs). An optimal Bayesian network, which incorporates correlations between taxa, was also performed [see Additional file 6: Figures S5 (genus) and 6 (OTUs)].

This analysis revealed that RA disease activity was positively correlated with *Micrococcus* and *Renibaterium* at the genus level, and with various OTUs belonging to *Pseudonocardia*, *Streptococcus*, and *Xanthomonadaceae* (Fig. 4 and Additional file 5: Figure S4). The level of BAL eosinophils was also correlated with clinical activity (Additional file 6: Figure S5). Conversely, an *unclassified Oxalobacteraceae* genus had a negative correlation with DAS28 score at the time of BAL sampling (Fig. 4). A modest association with erosive disease was observed with the presence and abundance of *Pseudonocardia* in the RA BAL (85% of erosive RA patients vs 23% of non-erosive RA; *P* = 0.019 Additional file 4: Figure S3B). Noticeably, levels of BAL autoantibodies (i.e., anti-CCP2 in the BAL) correlated positively with the genus *Megasphera* and *unclassified Comamonadaceae* (Fig. 4) as well as OTUs belonging to these taxa (Additional file 5: Figure S4).

Serum IgA anti-CCP antibodies had a positive significant correlation with relative abundance of BAL *Enhydrobacter* and *unclassified Bradyrhizobiaceae* (Fig. 4; *P* = 0.015 and 0.004, respectively) and also with OTUs related to these and other genera, including *Veillonella* and unclassified *Stramenopiles* (Additional file 5: Figure S4). Serum IgG anti-CCP2 antibodies associated with an OTU related to *unclassified Comamonadaceae* within the RA BAL. The number of ACPA fine specificities in serum (NEFS) showed a positive correlation only with the genus *Prevotella* (Fig. 4) and with OTUs related to *Stramenopiles* and Streptococcus (Additional file 5: Figure S4 and Additional file 7: Figure S6).

Finally, while neutrophil abundance in BAL positively correlated with the genus *Neisseria* and a related OTU, lymphocytes levels were associated with the genus TM-7 and OTUs belonging to the genus *Prevotella*.

## Discussion

The role of the lung microbiome as mediator of inflammation has only recently emerged. Novel relevant work in the field revealed that (a) the human distal airways harbor several bacterial species constituting a unique ecological community [14, 20–26] and (b) changes upon alveolar inflammation occur in the presence of a distinctive microbial pneumotype [14, 15]. Utilizing high-throughput 16S sequencing we show here, for the first time, that the lung microbial composition in patients with early-untreated RA and lung sarcoidosis have a high degree of similarity and are significantly different from those of healthy controls. Importantly, several microbial signatures were associated with the inflammatory phenotype observed in RA. These findings further support the hypothesis that changes occurring in the lung microbial/host interface might contribute to disease pathogenesis in early stages of RA development.

In fact, the overall lung bacterial community structure in RA patients harbors 40 % less OTUs than healthy individuals. This is in line with prior studies in early RA subjects reporting dysbiotic states at other mucosal sites [7, 8, 27]. Interestingly, this decreased taxa diversity is very similar to what we found in BAL samples of sarcoidosis patients (a well-characterized inflammatory disease of the lung parenchyma). Importantly, RA and sarcoidosis patients not only shared an overall decreased BAL microbial richness and diversity, but the taxa driving the dysbiotic process were remarkably similar. Indeed, most higher taxonomic clades that were underrepresented in RA BAL compared to healthy subjects were also diminished or absent in sarcoidosis samples, including the families *Burkholderiaceae*, *Actinomycetaceae*, and *Spirochaetaceae* and the genera *Actynomyces*, *Treponema*, and *Porphyromonas*. The high degree of similarity between the lung microbiome in RA and sarcoidosis, despite the fact that RA patients were more frequently smokers and significantly older, suggests the possibility that airway mucosal inflammation is a potential driver of lung dysbiosis independently of smoking and age in both diseases. Alternatively, it is conceivable that the inflammatory process present in both disease states might also have an impact on the lung microbiota community. Importantly, however, RA patients were enrolled early in the disease process, therefore minimizing the confounding effects of prolonged inflammatory states and/or immunomodulatory therapeutic consequences on the microbiome [24].

Despite these similarities between RA and sarcoidosis, few exceptions exist. *Pseudonocardia*, most notably, was one of the few genera found to be more abundant in RA BAL compared to healthy controls and also the one correlating with higher disease activity (OTU level) and erosive arthritis. *Pseudonocardia* is a known antifungal commensal microorganism [28] and a higher abundance of this taxon might rather reflect the presence of fungal organisms in the distal airways. Although fungi have been implicated in the pathogenesis of the SKG arthritis model [29], we did not assess for their presence in the current study. Efforts are underway to incorporate mycobiome analysis into our current and future studies.

Interestingly, the presence of the genus *Prevotella* in the RA BAL (and a *Prevotella*-related OTU) significantly correlated with levels of systemic RF (IgA) and the number of ACPA fine specificities. This is in line with previous studies describing the presence of this genus in the RA-associated oral mucosa and showing that *Prevotella nigrescens* can trigger arthritis in mice [30, 31]. However, despite previous speculations regarding the arthritogenic potential of other periodontopathic microorganisms (most notably *P. gingivalis*), we report here a general underrepresentation of several of these genera in the BAL, including not only *Actinomyces* and *Prevotella* but *Porphyromonas* as well. *P. gingivalis*, one of the *Porphyromonas* species, carries the enzyme petydil-arginine-deiminase (PAD), which is responsible for the translational modification of arginine residues into citrullinated peptides, ultimately leading to the generation of neo-epitopes recognized by ACPAs [27, 30, 32–34]. Although our a priori hypothesis was that we would find higher prevalence of *P. ginigvalis* in the BAL, presumably via microaspiration, the seemingly paradoxical lower relative abundance of *Porphyromonaceae phylum* compared to controls aligns with recent studies concluding that mucosal periodontal inflammation (but not *P. gingivalis* abundance per se) is associated with RA prevalence [33]. Whether this represents a true lack of association or a site-specific lack of correlation remains to be further elucidated.

Our results also confirm previous data showing that the distal airway compartment is not sterile and provide detailed characterization of the local bacterial taxa using high-throughput sequencing. Although the material studied here is a unique one (consisting of BAL of early-untreated RA patients with short symptom duration and no clinical signs of lung involvement), the relatively low number of participants is a limitation. Bronchoscopic sampling was restricted to only one site in the lower airways, thus regional variation could not be investigated. Additionally, no oral samples were concomitantly obtained, so the question of cross-contamination with oral secretions could not be evaluated. However, contamination with oropharyngeal microbiota, once a matter of great debate, has not been shown to be a major and frequent event that would preclude utilization of bronchoscopy to sample the lower airways [14, 15, 23, 35]. Further, although technical controls were included in our sequence, BAL samples were obtained prior to adapting our now standardized protocol that uses bronchoscopic environmental controls. This controls may also be of relevance for microbiome studies with low biomass samples [36, 37]. Although healthy subjects were significantly younger, age has not been found to be a major factor in other lung microbiome studies [14, 22, 26]. However, it is conceivable that an age-related decrease in bacterial clearance and/or a relatively less robust immune response to the antigenic load could potentially alter the composition of the lower airway microbiome. Finally, our study does not address whether these dysbiotic changes precede or are rather a consequence of the inflammatory process. Studies on preclinical disease state, although difficult to perform given the cohort characteristics and the invasiveness of bronchoscopy, will be required to address this issue.

## Conclusions

In summary, we demonstrate that RA is characterized by a state of distal airway dysbiosis similar to that seen in sarcoidosis, a well-characterized inflammatory lung disease. We further identify a lower relative abundance of several taxa and a concomitant disease specific overrepresentation of a *Pseudonocardia* OTU in RA. Future mechanistic insights into directionality and possible causation will most likely be dependent on well-characterized prospective human studies and data derived from in vivo experiments from animal models.

## Additional files

Additional file 1: Supplemental materials (DOCX 39 kb)
Additional file 2: Figure S1. Unweighted UniFrac distance metric was used to compare BAL microbial communities within and between groups. Healthy BAL community structure was significantly different from sarcoid (A) and RA (B). By contrast, sarcoidosis and RA groups revealed a closer relative relatedness of taxonomic composition (C). (JPEG 146 kb)
Additional file 3: Figure S2. Cladrogram showing significantly different BAL genus by LefSe. (A) RA vs healthy, (B) sarcoid vs healthy, and (C) RA vs sarcoid. Empty bars reflect LDA effect size for each genera. Colored bars show taxa relative abundance for indicated groups. (JPEG 527 kb)
Additional file 4: Figure S3. Relative abundance of *Pseudonocardia OTU* in RA vs healthy and sarcoidosis (A) and correlation between genus *Pseudonocardia* and erosive RA (B). (JPEG 91 kb)
Additional file 5: Figure S4. RA BAL operational taxonomic unit (OTU) correlations with local/systemic autoimmunity, inflammatory markers and clinical disease activity. The relative abundance of BAL taxa was assessed for correlations with disease activity score (DAS28), the levels of serum acute phase reactants and autoantibodies (including number of fine specificities), and BAL levels of anti-CCP2 and immune cells (%). The heat map shows the correlations between patient metadata and BAL microbiota at the OTU level. Circle sizes and color intensity represent the magnitude of correlation. Blue circles = positive correlations; red circles = negative correlations. CRP = C-reactive protein; ESR = erythrosedimentation rate; NFS = number of ACPA fine specificities (ISAC chip); NEFS = number of ELISA ACPA fine specificities (JPEG 232 kb)
Additional file 6: Figure S5. Optimal Bayesian network analysis at the genus level. Green circles = taxa; blue circles = metadata; black lines = positive correlations; blue lines = negative correlations. (JPEG 149 kb)
Additional file 7: Figure S6. Optimal Bayesian network analysis at the OTU level. Green circles = taxa; blue circles = metadata; black lines = positive correlations; blue lines = negative correlations. (JPEG 173 kb)

## Abbreviations

ACPA: Anti-citrullinated protein antibody
BAL: Bronchoalveolar lavage
CCP: Cyclic citrullinated peptide
DMARD: Disease-modifying anti-rheumatic drugs
RA: Rheumatoid arthritis
RNA: Ribonucleic acid

## References

[1] McInnes IB, Schett G (2011). The pathogenesis of rheumatoid arthritis. N Engl J Med.

[2] van de Sande MG (2011). Different stages of rheumatoid arthritis: features of the synovium in the preclinical phase. Ann Rheum Dis.

[3] Scher JU, Abramson SB (2011). The microbiome and rheumatoid arthritis. Nat Rev Rheumatol.

[4] Rehaume LM (2014). ZAP-70 genotype disrupts the relationship between microbiota and host, leading to spondyloarthritis and ileitis in SKG mice. Arthritis Rheumatol.

[5] Wu HJ (2010). Gut-residing segmented filamentous bacteria drive autoimmune arthritis via T helper 17 cells. Immunity.

[6] Scher JU (2015). Intestinal dysbiosis and potential consequences of microbiome-altering antibiotic use in the pathogenesis of human rheumatic disease. J Rheumatol.

[7] Scher JU (2013). Expansion of intestinal Prevotella copri correlates with enhanced susceptibility to arthritis. Elife.

[8] Zhang X (2015). The oral and gut microbiomes are perturbed in rheumatoid arthritis and partly normalized after treatment. Nat Med.

[9] Catrina AI, Ytterberg AJ, Reynisdottir G, Malmstrom V, Klareskog L (2014). Lungs, joints and immunity against citrullinated proteins in rheumatoid arthritis. Nat Rev Rheumatol.

[10] Chatzidionisyou A, Catrina AI (2016). The lung in rheumatoid arthritis, cause or consequence?. Curr Opin Rheumatol.

[11] Reynisdottir G (2015). Signs of immune activation and local inflammation are present in the bronchial tissue of patients with untreated early rheumatoid arthritis. Ann Rheum Dis.

[12] Willis VC (2013). Sputum autoantibodies in patients with established rheumatoid arthritis and subjects at risk of future clinically apparent disease. Arthritis Rheum.

[13] Reynisdottir G (2014). Structural changes and antibody enrichment in the lungs are early features of anti-citrullinated protein antibody-positive rheumatoid arthritis. Arthritis Rheumatol.

[14] Segal LN (2013). Enrichment of lung microbiome with supraglottic taxa is associated with increased pulmonary inflammation. Microbiome.

[15] Segal LN (2016). Enrichment of the lung microbiome with oral taxa is associated with lung inflammation of a Th17 phenotype. Nat Microbiol.

[16] Arnett FC (1988). The American Rheumatism Association 1987 revised criteria for the classification of rheumatoid arthritis. Arthritis Rheum.

[17] Olsen HH, Grunewald J, Tornling G, Skold CM, Eklund A (2012). Bronchoalveolar lavage results are independent of season, age, gender and collection site. PLoS One.

[18] Karimi R, Tornling G, Grunewald J, Eklund A, Skold CM (2012). Cell recovery in bronchoalveolar lavage fluid in smokers is dependent on cumulative smoking history. PLoS One.

[19] Hansson M (2012). Validation of a multiplex chip-based assay for the detection of autoantibodies against citrullinated peptides. Arthritis Res Ther.

[20] Charlson ES (2011). Topographical continuity of bacterial populations in the healthy human respiratory tract. Am J Respir Crit Care Med.

[21] Erb-Downward JR (2011). Analysis of the lung microbiome in the “healthy” smoker and in COPD. PLoS One.

[22] Morris A (2013). Comparison of the respiratory microbiome in healthy non-smokers and smokers. Am J Respir Crit Care Med.

[23] Bassis CM (2015). Analysis of the upper respiratory tract microbiotas as the source of the lung and gastric microbiotas in healthy individuals. MBio.

[24] Pragman AA, Kim HB, Reilly CS, Wendt C, Isaacson RE (2012). The lung microbiome in moderate and severe chronic obstructive pulmonary disease. PLoS One.

[25] Sze MA (2012). The lung tissue microbiome in chronic obstructive pulmonary disease. Am J Respir Crit Care Med.

[26] Lozupone C (2013). Widespread colonization of the lung by tropheryma whipplei in HIV infection. Am J Respir Crit Care Med.

[27] Scher JU, Abramson SB (2013). Periodontal disease, Porphyromonas gingivalis, and rheumatoid arthritis: what triggers autoimmunity and clinical disease?. Arthritis Res Ther.

[28] Sen R (2009). Generalized antifungal activity and 454-screening of Pseudonocardia and Amycolatopsis bacteria in nests of fungus-growing ants. Proc Natl Acad Sci U S A.

[29] Yoshitomi H (2005). A role for fungal {beta}-glucans and their receptor Dectin-1 in the induction of autoimmune arthritis in genetically susceptible mice. J Exp Med.

[30] Scher JU (2012). Periodontal disease and the oral microbiota in new-onset rheumatoid arthritis. Arthritis Rheum.

[31] de Aquino SG, Abdollahi-Roodsaz S, Koenders MI, van de Loo FA, Pruijn GJ, Marijnissen RJ, Walgreen B, Helsen MM, van den Bersselaar LA, de Molon RS, Avila Campos MJ, Cunha FQ, Cirelli JA, van den Berg WB (2014). Periodontal pathogens directly promote autoimmune experimental arthritis by inducing a TLR2- and IL-1-driven Th17 response. J Immunol.

[32] Pischon N (2008). Association among rheumatoid arthritis, oral hygiene, and periodontitis. J Periodontol.

[33] Mikuls TR (2014). Periodontitis and Porphyromonas gingivalis in patients with rheumatoid arthritis. Arthritis Rheum.

[34] Mikuls TR (2012). Porphyromonas gingivalis and disease-related autoantibodies in individuals at increased risk of rheumatoid arthritis. Arthritis Rheum.

[35] Dickson RP, Erb-Downward JR, Martinez FJ, Huffnagle GB (2016). The microbiome and the respiratory tract. Annu Rev Physiol.

[36] Segal LN, Dickson RP (2016). The lung microbiome in HIV. Getting to the HAART of the host-microbe interface. Am J Respir Crit Care Med.

[37] Salter SJ (2014). Reagent and laboratory contamination can critically impact sequence-based microbiome analyses. BMC Biol.

