# Supplementary material for: The lung microbiota in early rheumatoid arthritis and autoimmunity
Source: Microbiome. 2016 Nov 17;4:60. doi: 10.1186/s40168-016-0206-x (PMC5114783; doi:10.1186/s40168-016-0206-x)
Supplement: Additional file 1: — Supplemental materials (DOCX 39 kb) [file 40168_2016_206_MOESM1_ESM.docx]

**SUPPLEMENTARY MATERIAL**

**Material and Methods**

**Bronchoscopy and Retrieval of BAL**

To obtain BAL, the bronchoscope (Olympus) was wedged in a middle lobe bronchus, and 5 aliquots (50 ml each) of phosphate buffered saline (PBS) were instilled. The fluid was gently suctioned back to a bottle kept on ice. Dwell-time was kept to a minimum, in accordance with the European Society of Pneumology Task Group guidelines [1]. The bronchoscopy procedure and handling of the BAL have been previously described in detail [2, 3].

**DNA extraction and sequence analysis.** Whole unfractionated BAL samples were utilized. Samples were stored at −80 °C until processing. Extraction of bacterial DNA from the samples was performed (Qiagen). Technical negative controls included DNA free water processed in parallel with BAL samples (which include DNA isolation and library preparation) and elution buffer (from the DNA isolation kit). High-throughput sequencing of bacterial 16S rRNA gene amplicons encoding the V4 region (150bp read length, paired-end protocol) was performed using the MiSeq Illumina sequencer to define the microbiota composition. Reagent controls were sequenced and analyzed as quality controls. The obtained 16S rRNA sequences were analyzed using the Quantitative Insights into Microbial Ecology (QIIME) pipeline for analysis of community sequence data as published by us [4-9]. Briefly, reads were demultiplexed and quality filtered with default parameters using prinseq. Sequences were then clustered into operational taxonomic units (OTUs) using a 97% similarity threshold with USEARCH and the Greengenes 16S reference dataset and taxonomy [10]. None of the OTUs identified in the technical controls were dominant in any BAL sample. Thus, we did not perform any OTU subtraction from the data. To avoid bias due to the different number of sequences per sample, 3687 sequences (minimum number of sequences found in a given sample) were used per sample in order to define OTUs and taxonomic composition. We used the ade4 package in R to construct Principal Coordinate Analysis (PCoA) based on unweighted UniFrac distances [8, 11].

**ACPA assays**

Anti-CCP2 antibodies in the serum were detected using the enzyme-linked immunosorbent assay (ELISA) (Euro-Diagnostica AB, Sweden) according to manufacturer’s instructions. Serum samples were analyzed for specific ACPA IgGs using a custom-made peptide microarray based on the ImmunoCAP ISAC system (PhaDia) described in detail earlier [12].

**Statistical analysis.**  In order to identify differentially abundant bacterial taxa among the 3 groups, we applied the LefSe analytic method. LefSe is a biomarker-discovery approach based on an algorithm that first performs a nonparametric Kruskal-Wallis test in order to identify bacterial taxa whose relative abundance is significantly different between groups. Subsequently, LefSe applies Linear discriminant analysis (LDA) to those bacterial taxa identified as significantly different (at *P* < 0.05) and further assesses the effect size of each differentially abundant taxon. Only those taxa that obtain a log LDA score >2 were ultimately considered to discriminate between phenotypes. In addition, since multiple hypothesis testing is not considered in the LefSe analytic method, we further applied the Benjamini and Hochberg false discovery rate (FDR) test. Those bacterial taxa/OTUs showing a significant difference at *P* < 0.05 (Kruskal-wallis test) and a FDR q value < 0.1 were considered to be the main bacterial taxa differentiating between the three groups of samples. Subsequently, the Mann-Whitney U test was applied to define statistical significance between pair of groups. Only those taxa and OTUs with a relative abundance > 0.1% were analyzed, with the exception of the genera *Porphyromonas* (mean abundance =0.09%) that was included in the analysis for its known association with RA. At the OTU level, to diminish the number of multiple hypotheses, we only analyzed OTUs from those taxa that were found to be statistically significant among groups.

For cross-sectional analyses of baseline characteristics and comparison of diversity indexes between groups, differences were evaluated using the two-tailed Student’s *t*-test. The ANOSIM test was applied to the unweighted UniFrac distance matrix containing all analyzed samples in order to define if the overall structure of the microbiota was significantly different between the different groups. P values less than 0.05 were considered significant.

**Correlation analyses.** Spearman’s correlation analyses were used to assess correlations between taxa, anti-CCP levels/ACPA spreading, and inflammatory protein levels. Only taxa and OTUs present in at least 10% of the samples were analyzed. *P* values less than 0.05 were considered significant. Lower correlated variables (Spearman correlation coefficient (rho) absolute value < 0.5) were discarded and are not shown in the Figures.

**Bayesian networks.** At a first stage, an optimal Bayesian network structure was inferred through the “high climbing” algorithm implemented in the bnlearn R package[13]. To get that network, regularized inference was carried out by rejecting those relationships between nodes with associated Spearman’s correlation p-value greater than 0.05 or Spearman’s correlation coefficient absolute value lower than 0.5. Secondly, the concept of Markov blancket of nodes in Bayesian Networks was used in order to focus on a minimal subset of the network gathering only nodes associates with clinical variables together with their most neighboring nodes. That subnetwork is informative enough given that the Markov blanket of a node can be understood as the only knowledge needed to predict the behavior of that node.

**REFERENCES**

1. *Clinical guidelines and indications for bronchoalveolar lavage (BAL): Report of the European Society of Pneumology Task Group on BAL.* Eur Respir J, 1990. **3**(8): p. 937-76.

2. Olsen, H.H., et al., *Bronchoalveolar lavage results are independent of season, age, gender and collection site.* PLoS One, 2012. **7**(8): p. e43644.

3. Karimi, R., et al., *Cell recovery in bronchoalveolar lavage fluid in smokers is dependent on cumulative smoking history.* PLoS One, 2012. **7**(3): p. e34232.

4. Caporaso, J.G., et al., *QIIME allows analysis of high-throughput community sequencing data.* Nature methods, 2010. **7**(5): p. 335-6.

5. Edgar, R.C., *Search and clustering orders of magnitude faster than BLAST.* Bioinformatics, 2010. **26**(19): p. 2460-1.

6. Wang, Q., et al., *Naive Bayesian classifier for rapid assignment of rRNA sequences into the new bacterial taxonomy.* Applied and environmental microbiology, 2007. **73**(16): p. 5261-7.

7. Caporaso, J.G., et al., *PyNAST: a flexible tool for aligning sequences to a template alignment.* Bioinformatics, 2010. **26**(2): p. 266-7.

8. Lozupone, C., et al., *UniFrac: an effective distance metric for microbial community comparison.* The ISME journal, 2011. **5**(2): p. 169-72.

9. Segal, L.N., et al., *Enrichment of lung microbiome with supraglottic taxa is associated with increased pulmonary inflammation.* Microbiome, 2013. **1**(1): p. 19.

10. McDonald, D., et al., *An improved Greengenes taxonomy with explicit ranks for ecological and evolutionary analyses of bacteria and archaea.* ISME J, 2012. **6**(3): p. 610-8.

11. Dray, S.a.D., A.B., *The ade4 package: implementing the duality diagram for ecologists.* Journal of Statistical Software, 2007. **22**(4): p. 1-20.

12. Hansson, M., et al., *Validation of a multiplex chip-based assay for the detection of autoantibodies against citrullinated peptides.* Arthritis Res Ther, 2012. **14**(5): p. R201.

13. Scutari, M., *Learning Bayesian networks with the bnlearn R package.* arXiv preprint arXiv:0908.3817, 2009.
