# Supplementary figures and images for: The lung microbiota in early rheumatoid arthritis and autoimmunity
Source: Microbiome. 2016 Nov 17;4:60. doi: 10.1186/s40168-016-0206-x (PMC5114783; doi:10.1186/s40168-016-0206-x)

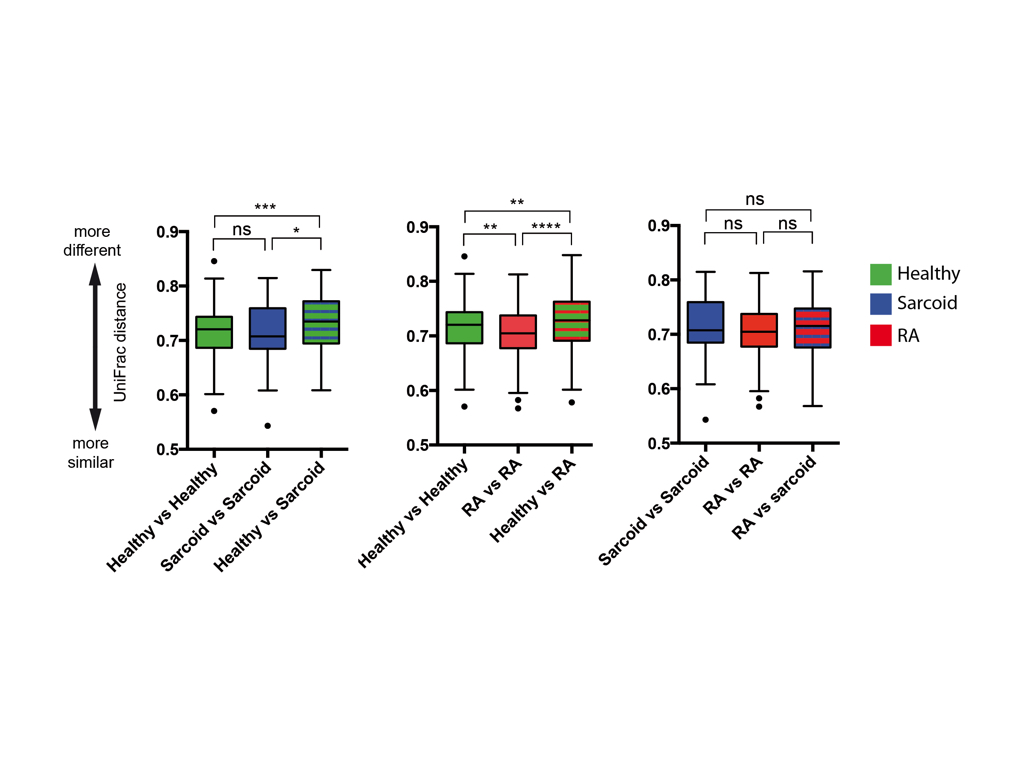

Supplement: Additional file 2: Figure S1. — Unweighted UniFrac distance metric was used to compare BAL microbial communities within and between groups. Healthy BAL community structure was significantly different from sarcoid (A) and RA (B). By contrast, sarcoidosis and RA groups revealed a closer relative relatedness of taxonomic composition (C). (JPEG 146 kb) [file 40168_2016_206_MOESM2_ESM.jpeg]

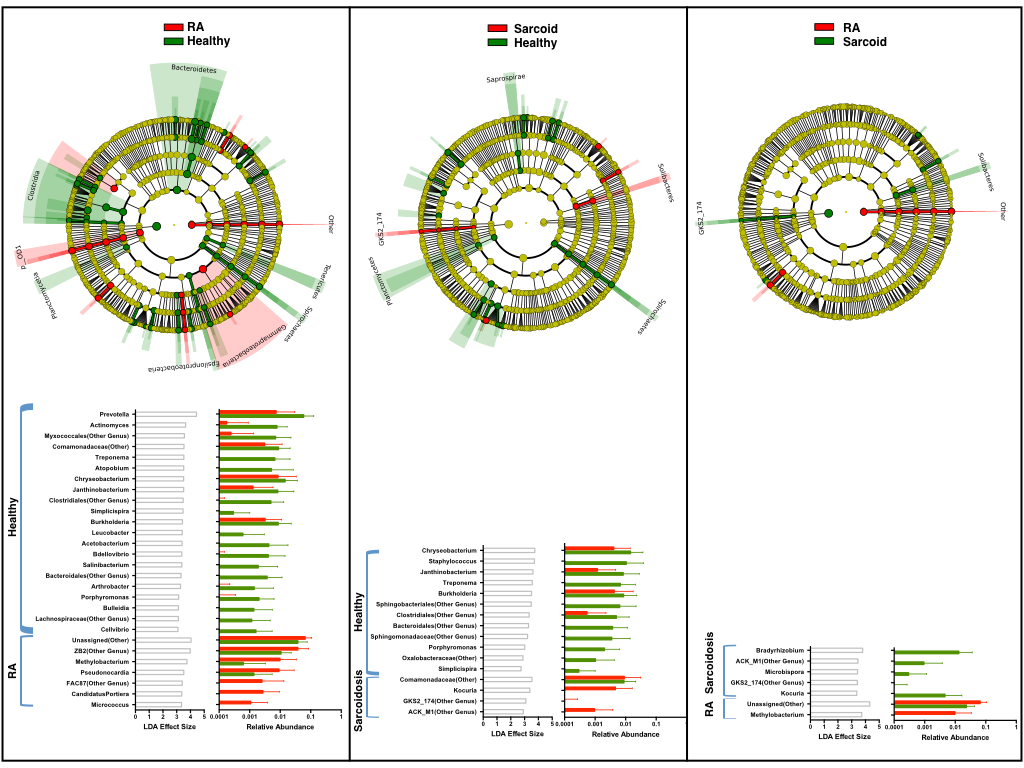

Supplement: Additional file 3: Figure S2. — Cladrogram showing significantly different BAL genus by LefSe. (A) RA vs healthy, (B) sarcoid vs healthy, and (C) RA vs sarcoid. Empty bars reflect LDA effect size for each genera. Colored bars show taxa relative abundance for indicated groups. (JPEG 527 kb) [file 40168_2016_206_MOESM3_ESM.jpeg]

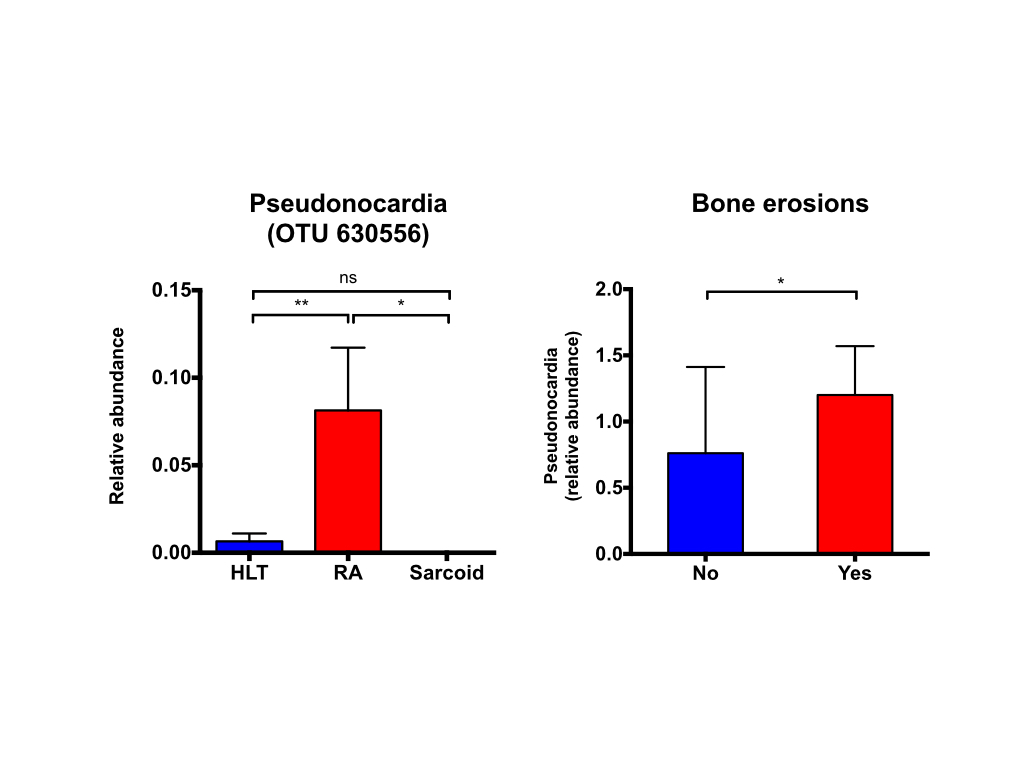

Supplement: Additional file 4: Figure S3. — Relative abundance of Pseudonocardia OTU in RA vs healthy and sarcoidosis (A) and correlation between genus Pseudonocardia and erosive RA (B). (JPEG 91 kb) [file 40168_2016_206_MOESM4_ESM.jpeg]

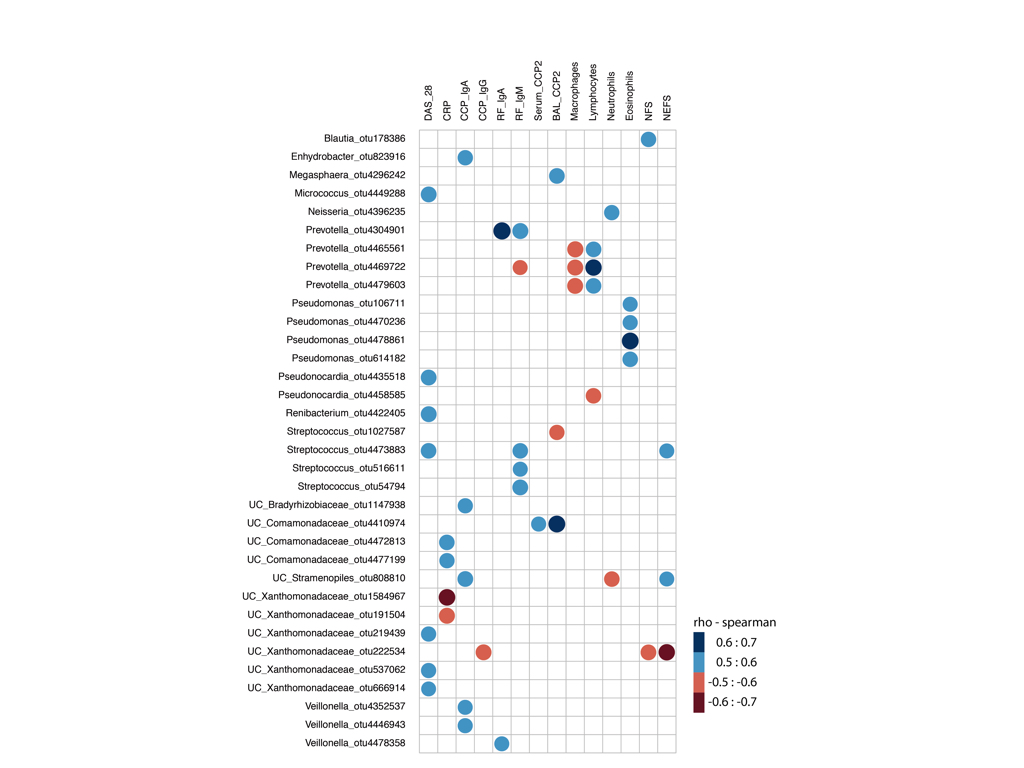

Supplement: Additional file 5: Figure S4. — RA BAL operational taxonomic unit (OTU) correlations with local/systemic autoimmunity, inflammatory markers and clinical disease activity. The relative abundance of BAL taxa was assessed for correlations with disease activity score (DAS28), the levels of serum acute phase reactants and autoantibodies (including number of fine specificities), and BAL levels of anti-CCP2 and immune cells (%). The heat map shows the correlations between patient metadata and BAL microbiota at the OTU level. Circle sizes and color intensity represent the magnitude of correlation. Blue circles = positive correlations; red circles = negative correlations. CRP = C-reactive protein; ESR = erythrosedimentation rate; NFS = number of ACPA fine specificities (ISAC chip); NEFS = number of ELISA ACPA fine specificities (JPEG 232 kb) [file 40168_2016_206_MOESM5_ESM.jpeg]

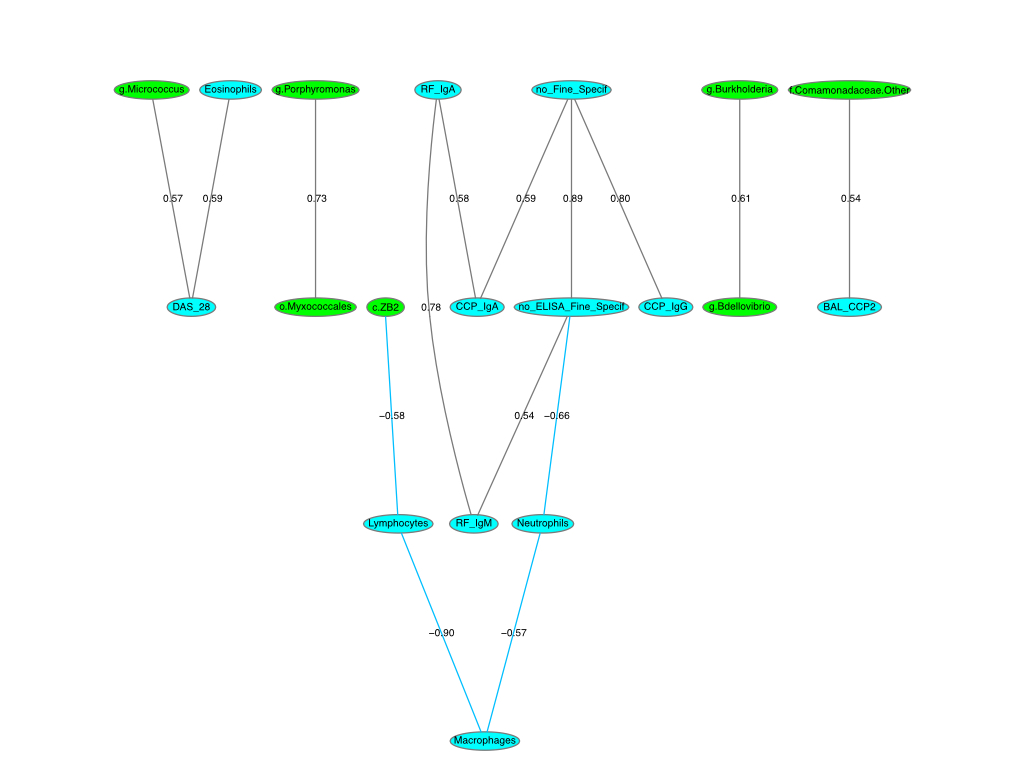

Supplement: Additional file 6: Figure S5. — Optimal Bayesian network analysis at the genus level. Green circles = taxa; blue circles = metadata; black lines = positive correlations; blue lines = negative correlations. (JPEG 149 kb) [file 40168_2016_206_MOESM6_ESM.jpeg]

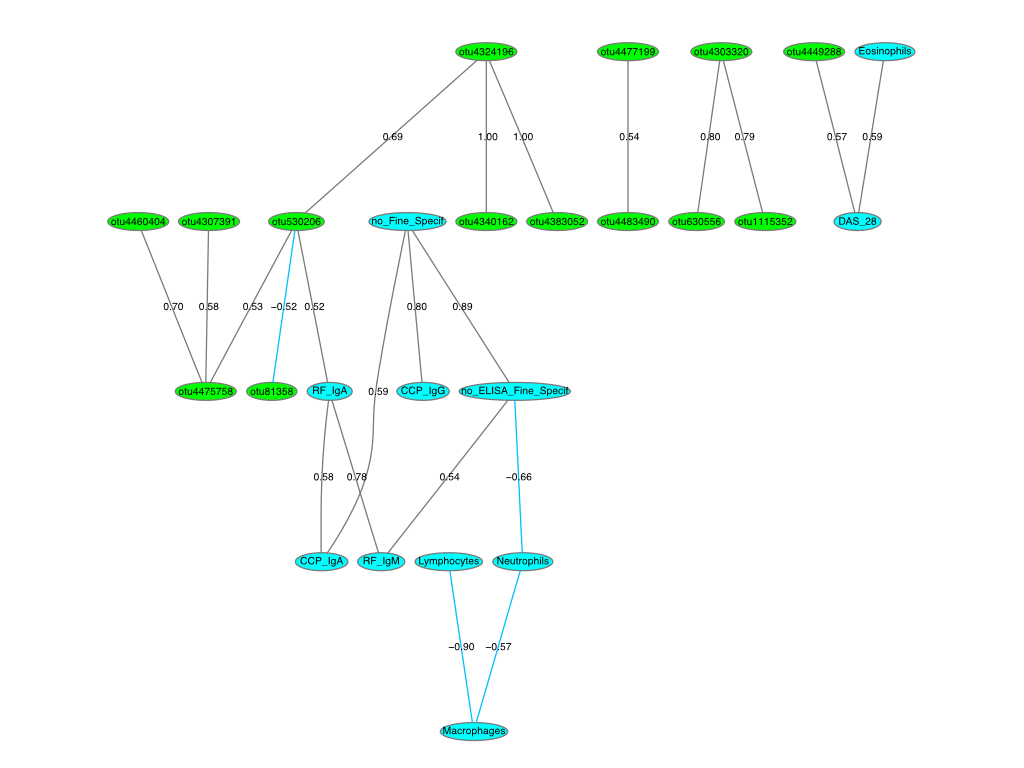

Supplement: Additional file 7: Figure S6. — Optimal Bayesian network analysis at the OTU level. Green circles = taxa; blue circles = metadata; black lines = positive correlations; blue lines = negative correlations. (JPEG 173 kb) [file 40168_2016_206_MOESM7_ESM.jpeg]
